# Combining molecular typing and spatial pattern analysis to identify areas of high tuberculosis transmission in a moderate-incidence county in Taiwan

**DOI:** 10.1038/s41598-017-05674-6

**Published:** 2017-07-14

**Authors:** Yih-Yuan Chen, Jia-Ru Chang, Chih-Da Wu, Yen-Po Yeh, Shiu-Ju Yang, Chih-Hao Hsu, Ming-Ching Lin, Ching-Fang Tsai, Ming-Shian Lin, Ih-Jen Su, Horng-Yunn Dou

**Affiliations:** 1 Department of Biochemical Science and Technology, National Chiayi University, Chiai-Yi, Taiwan; 2 National Institute of Infectious Diseases and Vaccinology, National Health Research Institutes, Zhunan, Miaoli, Taiwan; 3 Department of Forestry and Natural Resources, National Chiayi University, Chia-Yi, Taiwan; 4 The Center for Health and the Global Environment, Harvard T. H. Chan School of Public Health, Boston, MA USA; 5 Department of Medical Research, Ditmanson Medical Foundation, Chia-Yi Christian Hospital, Chia-Yi, Taiwan; 6 Department of Internal Medicine, Ditmanson Medical Foundation, Chia-Yi Christian Hospital, Chia-Yi, Taiwan; 7 Chang-Hua County Public Health Bureau, Changhua City, Taiwan

## Abstract

In total, 303 randomly selected clinical *Mycobacterium tuberculosis* (MTB) isolates from 303 patients (collected January to December 2012) in central Taiwan were examined. The major lineages found were Beijing (N = 114, 37.62%), Haarlem (N = 76, 25.08%) and East African–Indian (EAI) (N = 42, 13.86%). Notably, younger persons (≤30 years old) were 6.58 times more likely to be infected with a Beijing genotype compared to older persons (>70 years) (p < 0.05). Combining molecular typing methods and geographical information system (GIS) analysis, we uncovered a twofold higher incidence of Beijing strains in a hotspot area (33%) compared to non-hotspot areas (17%). By 24 MIRU-VNTR typing, persons in clustered groups were 1.96 times more likely to be infected with a Beijing strain compared with non-clustered persons, suggesting recent spread and emergence of MTB. Finally, we observed a trend in which TB incidence increased as the density/concentration of analyzed environmental factors increased, suggesting that environmental factors are associated with TB transmission; however, only population density was found to be significantly associated with increased risk of TB (p < 0.05). Molecular typing methods combined with spatial analysis suggest possible TB transmission. Early intervention to interrupt transmission may be most effective if targeted to hot zones of TB.

## Introduction

Tuberculosis (TB), an airborne infectious disease caused by *Mycobacterium tuberculosis* (MTB), is one of the deadliest communicable diseases worldwide. As estimated by the World Health Organization in 2013, there were 9 million new TB cases and 1.5 million TB deaths globally^1^. Although Taiwan has a low to medium incidence of TB cases compared to all other countries, TB is still a significant public health threat, especially in high-risk groups and regions^2, 3^. Therefore, early intervention and improved investigation of the contacts of TB cases are critical to interrupt transmission. Zelner *et al*. suggested that targeting the highest burden TB areas might be more effective than blanket screening and treatment^4^. In past decades, the transmission dynamics of TB in social networks has been analyzed based on molecular epidemiological methods, including restriction fragment length polymorphism, spoligotyping, and MIRU-VNTR (mycobacterial interspersed repetitive units – variable number of tandem repeats) typing^5–7^. Recently, molecular epidemiological techniques combined with graphically based surveillance have been applied to identify areas with ongoing TB transmission and as a new strategy to limit the spread of TB^4^.

We have undertaken long-term surveillance of MTB in central Taiwan (including collecting information on MTB genotypes, drug resistance phenotypes, and TB patient demographic characteristics) to ascertain associations between transmission dynamics and risk factors. In our earlier studies, we applied molecular epidemiological methods to analyze hundreds of randomly chosen MTB clinical isolates from patients in northern, southern and eastern regions of Taiwan. Those studies demonstrated that the Beijing lineage is the predominant MTB strain in Taiwan^3, 8–10^. Our molecular analyses also showed that the East African–Indian (EAI) and Haarlem strains are particularly prevalent in southern and eastern Taiwan, respectively^3, 9^. However, the transmission dynamics of prevalent MTB strains in central Taiwan are not well understood.

The purpose of the present study was twofold. First, we sought to determine the genotypes of MTB isolates present in central Taiwan. Second, we sought to evaluate the factors associated with increased risk of TB by using molecular methods combined with geographical information system (GIS) analysis. Interestingly, Lai *et al*. and Smith *et al*. reported that fine particle (PM2.5) and traffic-related air pollution, including nitrogen dioxide, nitrogen oxide and carbon monoxide, were associated with elevated TB risk^11, 12^. Therefore, in this study we also investigated environmental factors. We further sought to identify possible sites of TB transmission by using hotspot analysis in combination with GIS and molecular typing techniques. Our spatial analysis revealed possible TB transmission areas, which we believe may be helpful in setting public health policies to limit the spread of TB^4^.

## Materials and Methods

### Study setting

This retrospective study was conducted at the National Health Research Institutes in Taiwan. The study included 303 MTB samples (from 303 patients) randomly selected from 795 isolates collected during January 2012 to December 2012 at the Changhua Christian Hospital (CCH). The mycobacterium laboratory of CCH is the major laboratory in Changhua providing TB testing services not only to Changhua Christian Healthcare System (CCHC comprises one medical center CCH and three branch hospitals distributed evenly at different locations of the county and covering both urban and rural areas) hospitals, but also to 27 health centers and other primary care clinics in each township of this county. All bacteria isolates were confirmed by routine microscopy, culture and positive nitrate and niacin tests. All isolated 303 MTB strains were then genotyped by spoligotyping and 24-locus MIRU-VNTR typing. This study was approved by the Human Ethics Committee of the National Health Research Institutes, Taiwan (Code: EC1010804-E-R1).

### Genomic DNA extraction from mycobacterial cells

Mycobacterial genomic DNA extraction was performed as described previously^9^. Briefly, bacterial colonies were scraped from Lowenstein-Jensen medium and suspended in 100 μl distilled H_2_O. The samples were then incubated at 85 °C for 30 min. After centrifugation, the supernatant, containing DNA, was removed into a new tube and stored at −20 °C until use.

### Spoligotyping and spoligotype analysis

Spoligotyping was performed according to the manufacturer’s instructions (Isogen Bioscience BV., Maarsen, The Netherlands). The new isolates were assigned by using the SUITVITWEB database^13^ and Spotclust^14^.

### 24-locus MIRU-VNTR typing

The 24 loci (MIRU 4, MIRU 26, MIRU 40, MIRU 10, MIRU 16, MIRU 31, Mtub04, ETR C, ETR A, Mtub30, Mtub39, QUB-4156, QUB-11b, Mtub21, QUB-26, MIRU 2, MIRU 23, MIRU 39, MIRU 20, MIRU 24, MIRU 27, Mtub29, ETR B, and Mtub34) were selected and analyzed as described previously^15^. The resulting genotyping pattern of each isolated strain was used to create a digit-allelic profile.

### Drug susceptibility testing

Drug susceptibility tests were carried out according to the Clinical and Laboratory Standards Institute (CLSI) standard^16^. The tests were conducted by using the agar proportion method utilizing Middlebrook 7H10 agar supplemented individually with the following antibiotics: isoniazid (0.2 or 1 mg/L), rifampin (1 or 5 mg/L), ethambutol (5 or 10 mg/L), and streptomycin (5 or 10 mg/L).

### Spatial analysis

Residential addresses of each participant at the time of diagnosis were geocoded by ArcGIS and Taiwan Geospatial One-Stop Portal (TGOS), the official website of the Information Center, Ministry of the Interior of Taiwan for geospatial data management. Three spatial analyses were conducted: (1) the Global Moran’s I statistic was applied to measure the spatial autocorrelation of TB outbreak at the township scale; (2) Kernel Density Estimation (KDE) was applied to identify the geographic hotspots of TB in the Chang-hua area; and (3) the clustered MTB strains were overlaid with several thematic maps, including ones depicting population density, fine particulate matter (PM_2.5_) concentration, and residential, commercial and industrial areas, to visually assess potential spatial trends among human activities, air pollutants, and distribution of clustered TB strains. Also, townships (with their numbers of TB cases or numbers of clustered strains) were examined for differences in population density and the magnitude of three air pollutants (PM_2.5_, NO_2_, and CO).

Supplementary Fig. 1A,B show the spatial distribution of all isolated TB cases for the year 2012 (N = 795), and the randomly selected MTB strains examined in this study (N = 303), respectively. The consistent spatial distributions in those two figures confirm the representative nature of the sampled TB subjects in the study area and their suitability for empirical analysis.

**Figure 1:**
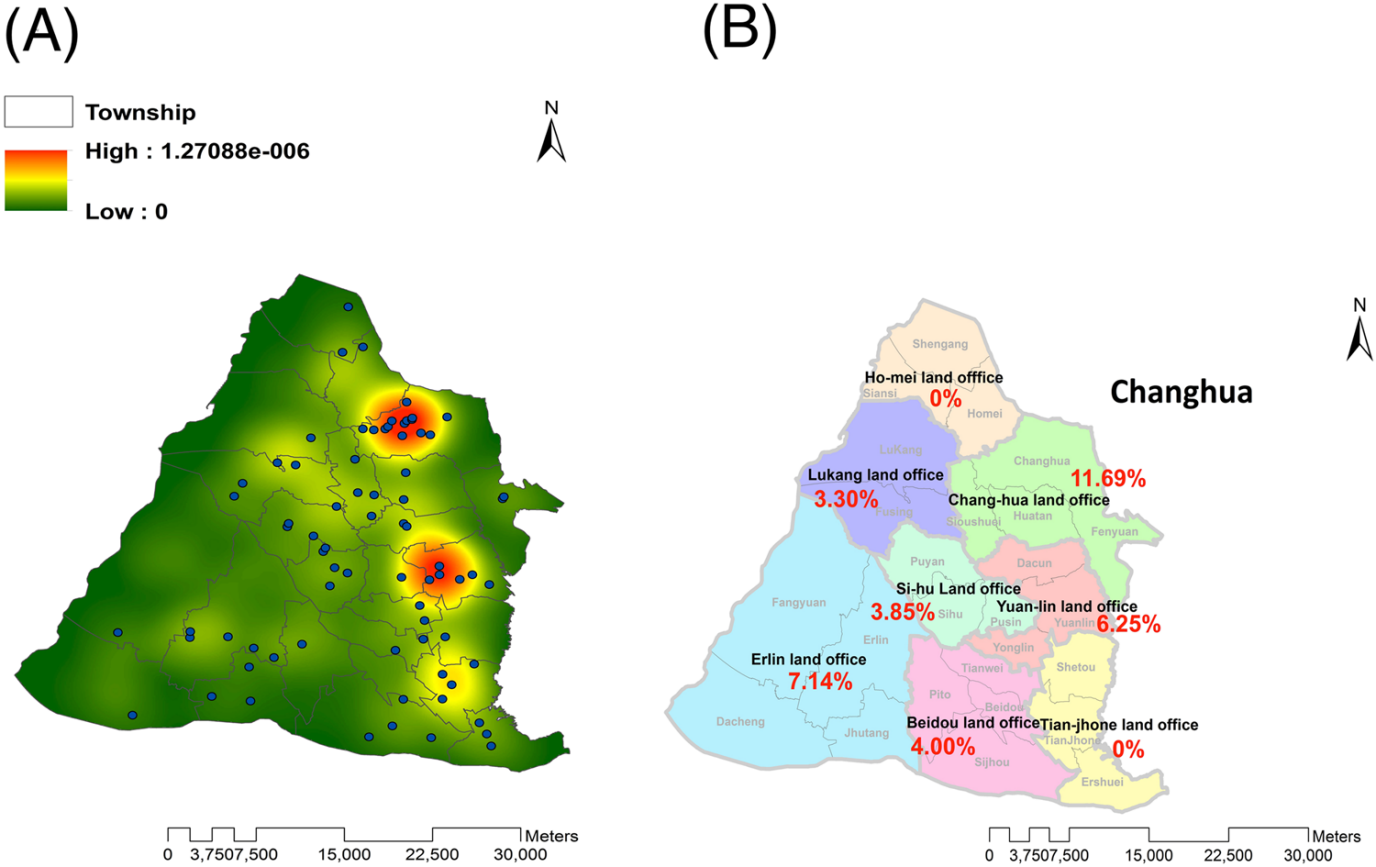
Spatial distribution of clustered MTB strains. (**A**) KDE maps were constructed based on GIS data representing the clustered MTB strain referral sites. (**B**) Distribution of clustered MTB strains was analyzed based on strain-clustering rates in each township. Esri ArcGIS 10.2 was used to create this figure (http://www.esri.com/software/arcgis).

### Kernel-density Estimation (KDE)

KDE is a tool for smoothing points in continuous space^17^. A smoothly curved surface is fitted over each point. The surface value is highest at the location of the point and diminishes with increasing distance from the point, reaching zero at the search radius distance from the point^18^. In our study, ArcMap GIS (version 10.2) and its Spatial Analyst Extension (ESRI Inc., Redland, CA) were used to generate the KDE maps with a search radius of 5 km. The distribution of MTB/clustered MTB strains was calculated using KDE methods.

### Spatial variability of PM_2.5_

Hourly ambient PM_2.5_ concentrations were measured by the Taiwan Environmental Protection Administration from January 1, 2012 to December 31, 2012. We used automatic monitoring data, which were available from 71 stationary sites covering the Main Island. A modified ordinary kriging adopted from Liao *et al*. (2006) was applied to determine the spatial estimation of PM_2.5_ levels of the whole island, and then the extent of the study area was extracted. ArcMap GIS (version 10.2) and its Geostatistical Analysts Extension (ESRI Inc., Redland, CA) were utilized to construct the semivariogram. The median value of cross-validated R-squared was 0.73^19^.

### Statistical analysis

Patient characteristics (gender, age) and the results of the sputum smear, chest X-ray, drug susceptibility test and MIRU-VNTR analysis were compared between Beijing- and non-Beijing-infected groups. Univariate regression analyses were performed to yield crude odds ratios and their 95% confidence intervals. Data management and analyses were carried out by using SAS/STAT software, version 9.3 for Windows (SAS Institute Inc., Cary, NC, USA).

## Results

### Demographic characteristics of TB patients

According to the TB registry database of the local health authority in Changhua county, a county with an intermediate incidence of TB (58.7 per 10^5^ in 2012), there were 856 reported pulmonary TB cases in 2012, 692 of which were confirmed by the sputum culture. The CCH TB laboratory provides nearly 70% of all TB testing services in the whole county each year. Thus, the 303 specimens randomly selected from 795 isolates collected at the CCH may therefore be treated as a representative sample of the total culture-positive pulmonary TB in the community. In total, 303 randomly selected clinical isolates from 303 patients in central Taiwan who were diagnosed with culture-confirmed TB (January to December 2012) were subjected to spoligotyping and MIRU-VNTR typing. There were 2.6 times as many isolates from men as from women (male: 71.95%, 218; female: 28.05%, 85). The most prevalent genotype was Beijing, identified in 114 isolates (37.62%), followed by Haarlem (76; 25.08%), EAI (42; 13.86%), T (37; 12.21%), Latin American–Mediterranean (LAM) (7; 2.31), and Manu_ancestor (7; 2.3%) (Table 1). The Beijing lineage was particularly prevalent in the ≤30-year-old age group (77.78%), although the sample size was small; its prevalence in three older age groups (31–50, 51–70 and >70) ranged from about 31% to 44%. In contrast, the prevalence of the Haarlem lineage was lowest in the ≤30-year-old age group (about 11%) and tended to increase with patient age (to a high of nearly 28%) (Table 1).

**Table 1:** Demographic characteristics of 303 tuberculosis patients from central Taiwan.

	Beijing	Haarlem	EAI	T	LAM	Manu_ancestor	Others
No. of isolates* (%)	114 (37.62)	76 (25.08)	42 (13.86)	37 (12.21)	7 (2.31)	7 (2.31)	20(6.60)
Gender							
Male	80 (36.70)	51 (23.39)	31 (14.22)	33 (15.14)	4 (1.83)	4 (1.83)	15 (6.88)
Female	34 (40.00)	25 (29.41)	11 (12.94)	4 (4.71)	3 (3.53)	3 (3.53)	5 (5.88)
Age(years, mean/median)	70/74.5	73/77.5	71/77.5	73/78	69/75	79.3/81	71/73.5
Age group (years)							
≤30	7 (77.78)	1 (11.11)	1 (11.11)	0 (0.00)	0 (0.00)	0 (0.00)	0 (0.00)
31–50	10 (31.25)	6 (18.75)	5 (15.63)	5 (15.63)	2 (6.25)	0 (0.00)	4 (12.50)
51–70	30 (44.12)	19 (27.94)	9 (13.24)	6 (8.82)	1 (1.47)	0 (0.00)	3 (4.41)
å 70	67 (34.54)	50 (25.77)	27 (13.92)	26 (13.40)	4 (2.06)	7 (3.61)	13 (6.70)

*One sample was collected from each patient.

### Characteristics of Beijing and non-Beijing strains isolated from TB patients

To better understand the clinical characteristics of patients infected with Beijing strains, the gender-, age-, sputum smear-, geographic location-, drug resistance and MIRU analysis-specific odds ratios were calculated (Table 2). There were no significant differences in gender, sputum smear, location and drug-resistance patterns between the Beijing and non-Beijing groups (Table 2). However, we found that persons in the youngest age group (≤30 years old) were 6.58 times more likely to be infected with a Beijing genotype compared with persons in the oldest age group (>70 years old) (Table 2). In addition, MIRU analysis revealed that patients in clustered groups were 1.96 times more likely to be infected with a Beijing strain compared with non-clustered patients, suggesting recent spread and emergence (Table 2).

**Table 2:** Characteristics of tuberculosis patients associated with *Mycobacterium* tuberculosis Beijing and non-Beijing strains.

	Bacterial lineage		Crude odds ratio (95% CI)
	Beijing; No. (%)	Non-Beijing; No. (%)	
Gender			
Male	80 (70.18)	138 (73.02)	0.8696 (0.520–1.45)
Female	34 (29.82)	51 (26.98)	Reference
Age			
≤30	7 (6.14)	2 (1.06)	6.582 (1.330–32.570)*
31–50	10 (8.77)	22 (11.64)	0.855 (0.383–1.910)
51–70	30 (26.32)	39 (20.63)	1.45 (0.826–2.534)
>70	67 (58.77)	126 (66.67)	Reference
Sputum smear			
Positive	62 (54.39)	93 (49.21)	1.31 (0.772–1.961)
Negative	52 (45.61)	96 (50.79)	Reference
Location			
Pulmonary	108 (94.74)	183 (96.83)	Reference
Extra-pulmonary	6 (5.26)	6 (3.17)	1.694 (0.533–5.385)
Antibiotic resistance pattern			
All sensitive	99 (86.84)	163 (86.24)	Reference
Monoresistance	2 (1.75)	12 (6.35)	0.2744 (0.006–1.252)
Polyresistance	13 (11.40)	13 (6.88)	1.646 (0.735–3.695)
Multidrug resistance	0 (0.00)	1 (0.53)	—
MIRU analysis			
Clustered	36 (31.58)	36 (19.05)	1.962 (1.147–3.354)*
Non-clustered	78 (68.42)	153 (80.95)	Reference

*Indicates that the OR is significant and p < 0.05.

### Using GIS tools to identify hot zones of TB transmission

The genotypes and clustering characteristics of isolated MTB strains can be analyzed by MIRU-VNTR typing. However, the hotspot areas of ongoing TB transmission cannot be identified using molecular methods. Therefore, we combined molecular and GIS analyses to identify geographical areas with ongoing TB transmission.

Figure 1A shows the distribution of clustered MTB strains calculated using the Kernel Density Estimation (KDE) method. Two geographical hotspots of MTB strains (red areas) surrounded by several regions of moderately intensive clustering (yellow areas) were observed in the west-central townships of Chang-hua and Yuan-lin. Figure 1B shows the strain-clustering rate in each geographical region based on molecular methods without GIS information. We noted a twofold higher incidence of Beijing strains in the two hotspot areas (33%, 12/24) compared to non-hotspot regions (17%; 7/35) (p = 0.087).

### Analysis of environmental risk factors associated with TB transmission

**Figure 2:**
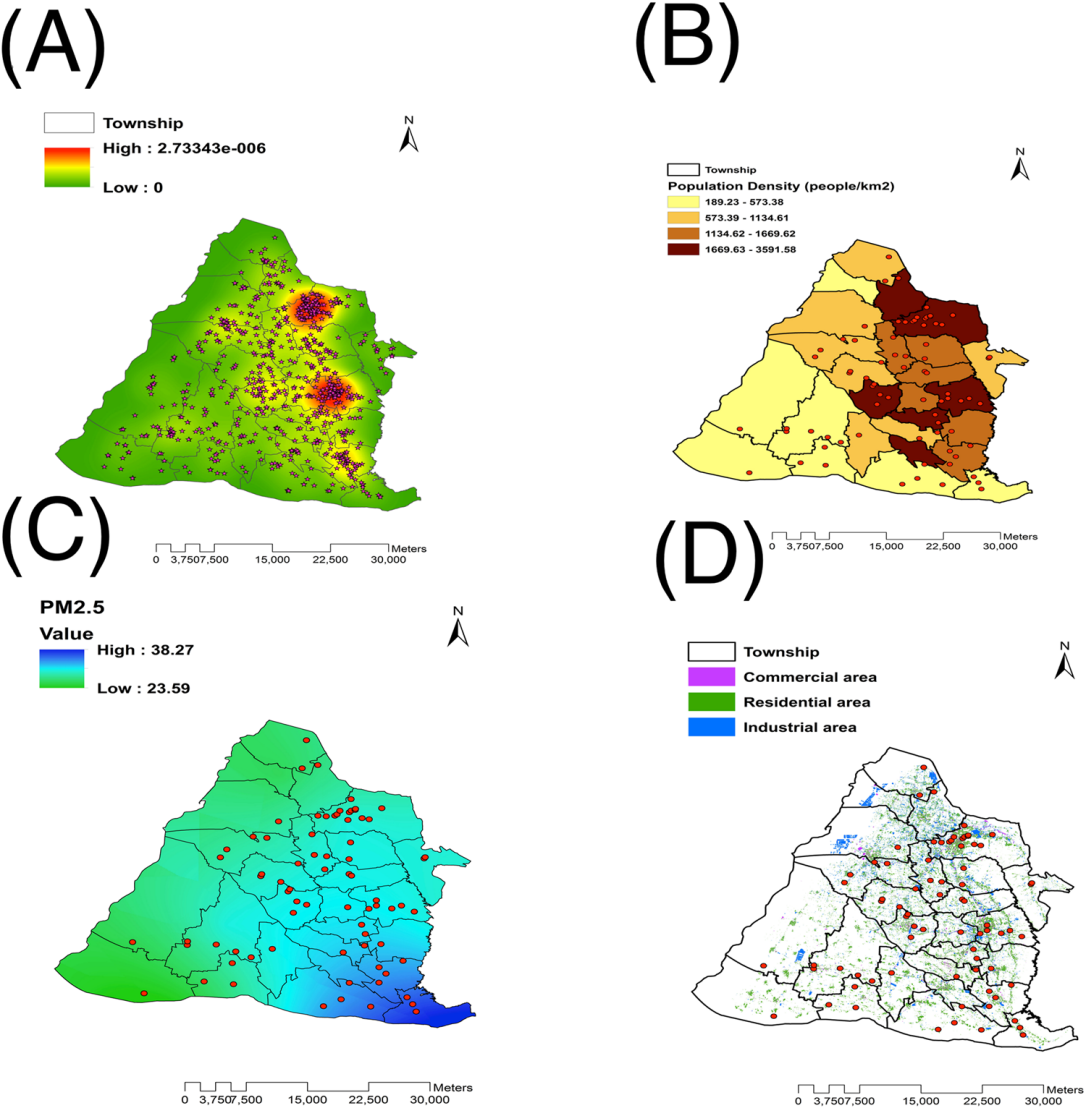
Distribution of Total MTB (**A**) and clustered strain (**B**, **C**, **D**) referral sites associated with (**B**) population density, (**C**) fine particle matter (PM_2.5_), and (**D**) human activity. Esri ArcGIS 10.2 was used to create this figure (http://www.esri.com/software/arcgis).

Lai *et al*. demonstrated that exposure to fine particles (PM_2.5_) and traffic-related air pollution (including nitrogen dioxide and carbon monoxide) was associated with increased risk of TB^12^. To investigate possible interactions between environmental factors and TB transmission, we examined the distribution of total TB cases/clustered strains associated with environmental factors, including population density, fine particulates, and human activity (Fig. 2).

Spatial analysis of TB cases revealed that MTB strains (including clustered strains) tended to group in townships with high population densities and residential areas (Fig. 2A,B and D). To clarify associations between environmental factors and TB transmission, we performed the Kruskal-Wallis H test. First, townships were classified into three categories based on the number of TB cases. Interestingly, TB incidence increased as the density/concentration of each analyzed environmental factor increased, suggesting that environmental factors are associated with TB transmission (Table 3). Nevertheless, only population density was significantly associated with increased risk of TB (Table 3; p = 0.046). No significant association between clustered strains and environmental factors was found (data not shown).

**Table 3:** Analysis of environmental factors for TB transmission by using the Kruskal-Wallis H test.

	TB case number	n	Mean rank	Chi-square statistic	p-value
Population density	fewest	9	8.56		
	mid	9	15	6.161	0. 046*
	most	8	17.38		
PM_2.5_	fewest	9	12.56		
	mid	9	13.89	0.214	0.899
	most	8	14.13		
NO_2_	fewest	9	9.11		
	mid	9	15.61	4.549	0.103
	most	8	16.06		
CO	fewest	9	11.56		
	mid	9	13.67	1.133	0.568
	most	8	15.50		

*p < 0.05.

## Discussion

To our knowledge, this is the first epidemiological study to combine GIS and molecular methods to investigate risk factors of TB transmission in Taiwan. Our results showed that younger persons (≤30 years old) were 6.58 times more likely to be infected with a Beijing genotype compared to older persons (>70 years old). Notably, hotspot regions have a twofold higher incidence of Beijing lineages (33%) compared to non-hotspot regions (17%). Based on the results of MIRU-VNTR typing, people in clustered groups were 1.96 times more likely to be infected with a Beijing strain compared with persons not in clustered groups. Taken together, our results suggest that targeting high-burden areas might be a good strategy to limit the spread of TB.

The Beijing genotype was the predominant genotype identified in central Taiwan, as well as in the two hotspot regions. This result coincides with those of previous studies in Taiwan and globally, in which Beijing strains, with their high transmissibility, are often associated with major TB outbreaks^8, 10, 20–22^. A recent study hypothesized that Beijing strains originated in Guangxi province in the south of China^23^, and they are the dominant MTB strains not only in China (up to 90%), but also in nearby countries, including South Korea (up to 97.1%), Japan (up to 80%%), Thailand (56%), Vietnam (up to 55.4%), Singapore (45.8%) and Myanmar (31.9%)^21, 22, 24–30^. The reasons for the success of Beijing strains remain controversial, but could include a variety of factors such as positive selection due to worldwide BCG vaccination coverage, population migration, and ineffective treatment, leading to disease transmission^22, 31^.

The patients in our cohort were, on average, older (70.0 years), and we found a trend of decreasing prevalence of Beijing genotype infections with age (Table 1). Moreover, Beijing is more common among young persons (≤30 years old; OR 6.58) and in clustered groups (OR 1.962), suggesting a tendency to spread in central Taiwan (Tables 1 and 2). In addition, as already noted, GIS results revealed a twofold higher incidence of Beijing lineages in hotspot areas (34%) compared to non-hotspot regions (17%), supporting increased transmission of Beijing strains in central Taiwan. The higher incidence of Beijing genotypes in hotspot areas may reflect the fact that young and middle-aged patients work and study in Chung-hua and Yuan-lin cities. These new results coincide with our previous findings in southern Taiwan and those reported by Buu *et al*. in Vietnam^32, 33^. In southern Taiwan, the percentage of Beijing-infected patients in young age groups increased monotonically from 3.9% in 2006 to 9.3% in 2008, but no such trend was observed for any non-Beijing genotype^33^. Apart from the Beijing genotype, the other major lineage found in central Taiwan was the Haarlem genotype (25.08%). Results of previous studies by our group revealed that the Haarlem lineage was predominant in eastern Taiwan, particularly in aborigines^2, 3^. In central Taiwan, most aborigines live in Nan-tou County, adjacent to Chang-hua city. A previous retrospective study conducted at Pulli Christian Hospital (located in Nan-tou County) demonstrated that Haarlem genotypes were the predominant MTB lineage^2^. Therefore, a high prevalence of Haarlem strains isolated in this study may be due to population migration and close interpersonal contact in those regions.

Interestingly, Lai *et al*. suggested that ambient air pollution is associated with increased risk of TB^12^. Similarly, Smith *et al*. suggested a potential association between long-term exposure to particulate matter and TB^11^. Notably, Rivas-Santiago *et al*. demonstrated that exposure of A549 cells, a human type II alveolar epithelial cell, to air pollution particulate matter induced cellular senescence, downregulation of beta-defensin (HBD-2 and HBD-3) expression, and impaired their ability to control MTB intracellular growth^34^. In the present study, associations between TB incidence and exposure to ambient air pollution, including fine particle matter (PM_2.5_), carbon monoxide and nitrogen dioxide, were evaluated. Our results coincide with those of previous studies, in which TB incidence increased as the density/concentration of the analyzed environmental factors increased; however, only population density was found to be significantly associated with increased risk of TB (p < 0.05). In developing countries, outdoor air pollution is a major problem and the people living in these regions experience long-term exposure to high levels of air pollutants. Taken together, these results suggest further investigation in the field of air pollution and TB control is warranted.

Our study has several limitations. The study was a retrospective one. Therefore, the effect on the results caused by missing data (e.g. host factors, including chronic diseases and genetics) is unknown. Also, the sample size for the oldest age group (>70 years) was small. We hope to initiate a prospective collection of patient information and MTB strains to further clarify associations between MTB transmission and risk factors such as environmental factors.

Despite these limitations, our study combined GIS and molecular epidemiological analyses to attempt to identify possible places of MTB transmission, with the idea that targeting high-burden areas might be an effective way to limit the spread of TB [2]. We observed a trend in which the numbers of TB cases increased as the density/concentration of the analyzed environmental factors increased. Taken together, our spatial analysis combined with molecular epidemiological methods has provided further insight into TB transmission dynamics in west-central Taiwan, and the approach should be considered as a new control policy in TB.

## Electronic supplementary material

Supplementary data
