# Supplementary material for: Combining molecular typing and spatial pattern analysis to identify areas of high tuberculosis transmission in a moderate-incidence county in Taiwan
Source: Sci Rep. 2017 Jul 14;7:5394. doi: 10.1038/s41598-017-05674-6 (PMC5511213; doi:10.1038/s41598-017-05674-6)
Supplement: Supplementary file 1 — Supplementary data [file 41598_2017_5674_MOESM1_ESM.pdf]

# Combining molecular typing and spatial pattern analysis to identify areas of high tuberculosis transmission in a moderate-incidence county in Taiwan

Yih-Yuan Chen[a], Jia-Ru Chang[b], Chih-Da Wu[c], Yen-Po Yeh[f], Shiu-Ju Yang[b], Chih-Hao Hsu[b], Ming-Ching Lin[b], Ching-Fang Tsai[d], Ming-Shian Lin[e] ,Ih-Jen Su[b] and Horng-Yunn Dou[b#]

[a]Department of Biochemical Science and Technology, National Chiayi University, Chia-Yi, Taiwan

[b]National Institute of Infectious Diseases and Vaccinology, National Health Research Institutes, Zhunan, Miaoli, Taiwan

[c]Department of Forestry and Natural Resources, National Chiayi University, Chia-Yi, Taiwan; The Center for Health and the Global Environment, Harvard T. H. Chan School of Public Health, Boston, MA, USA

[d]Department of Medical Research, Ditmanson Medical Foundation Chia-Yi Christian Hospital, Chia-Yi, Taiwan

[e]Department of Internal Medicine, Ditmanson Medical Foundation Chia-Yi Christian Hospital, Chia-Yi, Taiwan

[f]Chang-Hua County Public Health Bureau, Taiwan

[#]Corresponding author

Horng-Yunn Dou, Ph.D.,

E-mail address: hydou@nhri.org.tw

Mailing address: National Institute of Infectious Diseases and Vaccinology, National Health Research Institutes, Taiwan

No.35, Keyan Road, Zhunan Town, Miaoli County 350, Taiwan, ROC.

Tel: 886-37-246166 ext 35529   Fax: 886-37-583009

**Key words:** *Mycobacterium tuberculosis*, geographical information system (GIS), Beijing lineage

Running title: Spatial analysis of high TB transmission

## Figure legends

**Supplementary Figure 1.** Spatial distribution of TB cases in the study sites. (A) Total TB cases. (B) Random samples of molecularly analyzed TB cases. Esri ArcGIS 10.2 was used to create this figure (http://www.esri.com/software/arcgis).

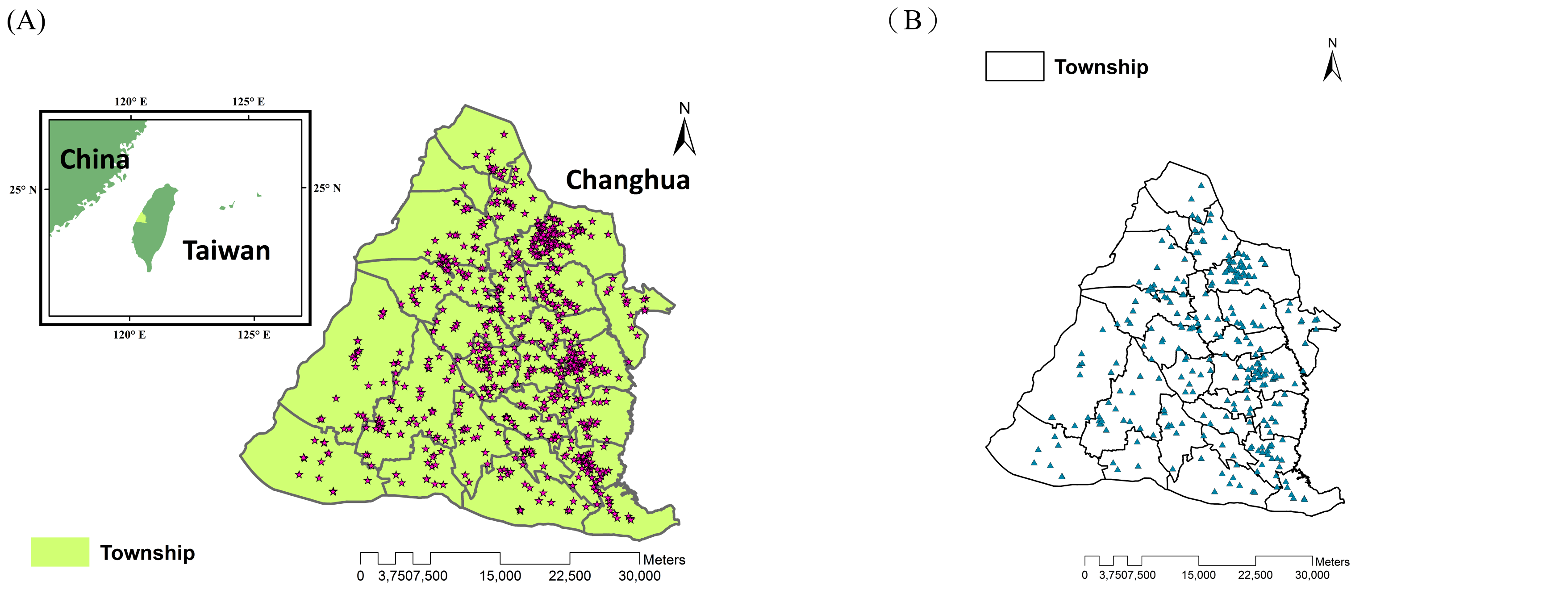
(A)
120° E
125° E
25° N
China
Taiwan
Changhua
N
Township
Meters
0
3,7507,500
15,000
22,500
30,000
(B)
Township
N
Meters
0
3,7507,500
15,000
22,500
30,000
